# Protection and Repair of the Nigrostriatal Pathway with Stem-Cell-Derived Carotid Body Glomus Cell Transplants in Chronic MPTP Parkinsonian Model

**DOI:** 10.3390/ijms24065575

**Published:** 2023-03-14

**Authors:** Javier Villadiego, Ana B. Muñoz-Manchado, Verónica Sobrino, Victoria Bonilla-Henao, Nela Suárez-Luna, Patricia Ortega-Sáenz, Ricardo Pardal, José López-Barneo, Juan J. Toledo-Aral

**Affiliations:** 1 Instituto de Biomedicina de Sevilla (IBiS), Hospital Universitario Virgen del Rocío/CSIC/Universidad de Sevilla, 41013 Sevilla, Spain; 2 Departamento de Fisiología Médica y Biofísica, Facultad de Medicina, Universidad de Sevilla, 41009 Sevilla, Spain; 3 Centro de Investigación Biomédica en Red sobre Enfermedades Neurodegenerativas (CIBERNED), 28029 Madrid, Spain; 4 Unidad de Biología Celular, Departamento de Anatomía Patológica, Biología Celular, Histología, Historia de la Ciencia, Medicina Legal y Forense y Toxicología, INiBICA (Instituto de Investigación e Innovación Biomédica de Cádiz), Universidad de Cádiz, 11003 Cádiz, Spain; 5 Instituto Maimónides de Investigación Biomédica de Córdoba, Departamento de Biología Celular, Fisiología e Inmunología, Universidad de Córdoba, 14004 Córdoba, Spain

**Keywords:** Parkinson’s disease, cell therapy, carotid body stem cells, neuroprotection, neurorestoration

## Abstract

Antiparkinsonian carotid body (CB) cell therapy has been proven to be effective in rodent and nonhuman primate models of Parkinson’s disease (PD), exerting trophic protection and restoration of the dopaminergic nigrostriatal pathway. These neurotrophic actions are mediated through the release of high levels of glial-cell-line-derived neurotrophic factor (GDNF) by the CB transplant. Pilot clinical trials have also shown that CB autotransplantation can improve motor symptoms in PD patients, although its effectiveness is affected by the scarcity of the grafted tissue. Here, we analyzed the antiparkinsonian efficacy of in vitro-expanded CB dopaminergic glomus cells. Intrastriatal xenografts of rat CB neurospheres were shown to protect nigral neurons from degeneration in a chronic MPTP mouse PD model. In addition, grafts performed at the end of the neurotoxic treatment resulted in the repair of striatal dopaminergic terminals through axonal sprouting. Interestingly, both neuroprotective and reparative effects induced by in vitro-expanded CB cells were similar to those previously reported by the use of CB transplants. This action could be explained because stem-cell-derived CB neurospheres produce similar amounts of GDNF compared to native CB tissue. This study provides the first evidence that in vitro-expanded CB cells could be a clinical option for cell therapy in PD.

## 1. Introduction

Parkinson’s disease (PD) is characterized by the progressive death of dopaminergic substantia nigra pars compacta (SNpc) neurons projecting to the striatum. The degeneration of nigral neurons results in the depletion of dopamine (DA) in the striatum, contributing to the typical motor symptoms of the disease. Current PD pharmacological therapies aim at increasing the striatal DA input through the administration of levodopa (a DA precursor), DA receptor agonists or inhibitors of DA degradation [1,2]. Furthermore, the transplantation of DA-secreting cells to increase the striatal DA content in PD patients has been intensively investigated. Among the different dopaminergic tissues tested, the best clinical benefit was obtained with allograft of human fetal ventral mesencephalic tissue (hfVM) in open-label trials [3,4,5]. However, two double-blind controlled trials questioned the clinical efficacy of this procedure, and showed the appearance of dyskinesias in some grafted patients [6,7]. The lack of a clinical improvement and the side effects observed in the double-blind controlled trials were attributed to methodological issues, such as patient selection, hfVM tissue preparation and implantation and the immunosuppressive regime applied post-transplantation [8,9]. Recently, a new open-label study, where these methodological issues have been strictly standardized, was designed to re-evaluate the clinical efficacy of hfVM grafts [10,11]. In addition, other antiparkinsonian cell-based therapies using midbrain dopaminergic progenitors generated from embryonic stem cells or induced pluripotent stem cells (iPSC) are currently being tested in clinical trials [12,13].

The carotid body (CB) is an alternative dopaminergic tissue used in antiparkinsonian cell-therapy [14]. The CB is a bilateral organ, located in the carotid bifurcation, that contains neural crest-derived highly dopaminergic glomus cells, which act as oxygen sensors that release dopamine in response to hypoxemia [15]. The intrastriatal transplantation of dopaminergic CB glomus cells was shown to promote significant recovery in rodent and nonhuman primate PD models [16,17,18,19,20]. Despite the high dopamine content of CB glomus cells, recovery induced through CB grafts is believed to be mainly mediated by the release of the glial-cell-line-derived neurotrophic factor (GDNF), which exerts neurotrophic protection and restorative effects on the nigrostriatal pathway [18,19,21]. Due to unilateral surgical CB resection having no significant side effects [22], two pilot clinical trials evaluated the safety and clinical efficacy of CB autotransplantation, showing that it can induce a clinical improvement in PD patients [23,24]. However, the small size of the CB and the potential damage of this organ in PD patients appear to be important limitations for the clinical outcome of CB autotransplantation [24,25,26]. During recent years, our laboratory has described and characterized a population of neural crest-derived stem cells in the adult CB. These neural stem cells are the sustentacular or type II cells in the organ, which, upon exposure to hypoxia, are converted into nestin^+^-proliferating progenitors and differentiate into new dopaminergic glomus cells. Furthermore, an in vitro culture of neural CB stem cells generates clonal cell colonies (named neurospheres) composed of a core of proliferating progenitors surrounded by blebs of differentiated dopaminergic CB glomus cells [27,28,29,30].

In this work, as proof of concept, we tested whether stem-cell-derived dopaminergic CB glomus cells exert neurotrophic protection to the damaged nigrostriatal pathway and, thus, ameliorate experimental parkinsonism. We showed that in vitro cultured CB neurospheres produced similar amounts of the dopaminotrophic factor GDNF than native CB tissue, and that GDNF expression was maintained after a striatal transplantation. Furthermore, intrastriatal grafts of CB neurospheres induced neuroprotective and restorative actions in the nigrostriatal pathway of chronic MPTP parkinsonian mice. Remarkably, the neurotrophic actions exerted by in vitro-expanded dopaminergic CB glomus cells were similar to those previously reported for CB tissue [19,31,32,33,34]. This study suggests that stem-cell-derived CB glomus cells could be a potential clinical option for cell therapy in PD.

## 2. Results

### 2.1. Histological Analysis and GDNF Expression of CB Stem-Cell-Derived Neurospheres

**Figure 1:**
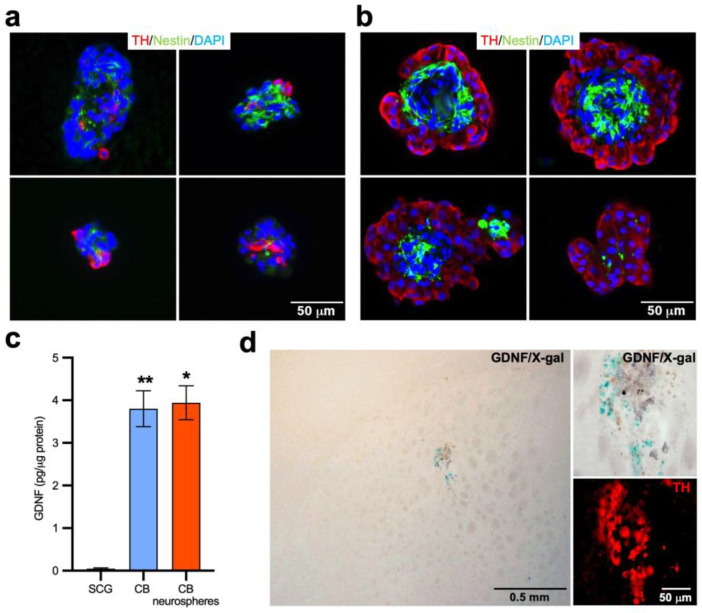
Characteristics of CB stem-cell-derived neurospheres. (**a**,**b**) High-magnification images of four representative examples of mouse (**a**) and rat (**b**) CB stem-cell-derived neurospheres after TH (red), nestin (green) and nuclear DAPI (blue) immunofluorescence. Note the presence of blebs with abundant TH^+^ glomus cells in the rat CB neurospheres. (**c**) GDNF content, expressed in pg/μg of total protein, of rat superior cervical ganglion (*n* = 7; SCG), native CB tissue (*n* = 6) and CB neurospheres (*n* = 3). (**d**) GDNF expression in intrastriatally grafted CB neurospheres from GDNF/X-gal heterozygous mice. The high magnification images in the right panels show the coincidence between the GDNF/X-gal (blue) and TH (red) fluorescence signals, clearly demonstrating the GDNF expression on grafted TH^+^ glomus cells. In c, the Kruskal–Wallis test and post hoc Dunn’s test were performed. * *p* < 0.05; ** *p* < 0.01, in respect to SCG.

Adult CBs contain quiescent multipotent neural stem cells that are activated under sustained hypoxemia, inducing organ growth. These neural stem cells have been identified as sustentacular type II CB cells, which, under hypoxia, change their quiescent GFAP^+^ state to a proliferative nestin^+^ phenotype and, thereafter, differentiate into mature dopaminergic CB glomus cells [27]. Moreover, CB neural stem cells form clonal colonies in vitro that resemble CB glomeruli, with a core of nestin^+^ progenitors surrounded by blebs of mature TH^+^ glomus cells [27,28,35]. Here, we assessed the in vitro culture of both mouse and rat CB neurospheres. As previously reported [36], the structure of CB-derived neurospheres is qualitatively similar between both rodent species, presenting proliferative nestin^+^ progenitors and differentiated TH^+^ glomus cells (Figure 1a,b). However, rat CB neurospheres are larger, and showed a higher number of differentiated TH^+^ glomus cells in the blebs than mouse CB neurospheres (Figure 1b). Because the recovery induced through CB cell therapy in parkinsonian mice being mainly due to the release of GDNF by the striatal graft [19], we analyzed the amount of the dopaminotrophic factor produced by rat CB neurospheres in comparison with other tissues. GDNF was measured using a specific enzyme-linked immunosorbent assay (ELISA) in the rat superior cervical ganglion (SCG), which does not express GDNF [21], native CB or CB neurospheres. As shown in Figure 1c, the CB neurospheres produced high levels of GDNF, similar to those of the native CB tissue. Moreover, we tested whether the ability to produce GDNF by stem-cell-derived CB glomus cells was maintained after an intrastriatal transplantation. Mouse CB neurospheres cultures were performed from heterozygous GDNF/lacZ animals (expressing beta-galactosidase under the control of GDNF promoter) and transplanted into the striatum of wild-type mice (*n* = 5). In these grafts, the cells expressing GDNF can be labelled with characteristic X-gal staining [19,21]. As indicated in Figure 1d, the intrastriatal transplants of the GDNF/lacZ CB neurospheres showed intense X-gal staining with numerous cells expressing GDNF. Remarkably, the combination of X-gal staining with TH immunofluorescence revealed a coincidence between X-gal deposits and the area covered with TH^+^ cells, indicating that stem-cell-derived dopaminergic CB glomus cells expressed high levels of GDNF after the striatal transplantation. Overall, these data clearly showed that in vitro-expanded CB glomus cells presented the same properties (a mature dopaminergic phenotype and a high GDNF expression even after an intracerebral transplantation) as native CB glomus cells. Therefore, our findings support the use of CB-derived neurospheres in antiparkinsonian cell therapy.

### 2.2. Protective and Restorative Effects of Stem-Cell-Derived CB Glomus Cell Therapy on the Nigrostriatal Pathway

**Figure 2:**
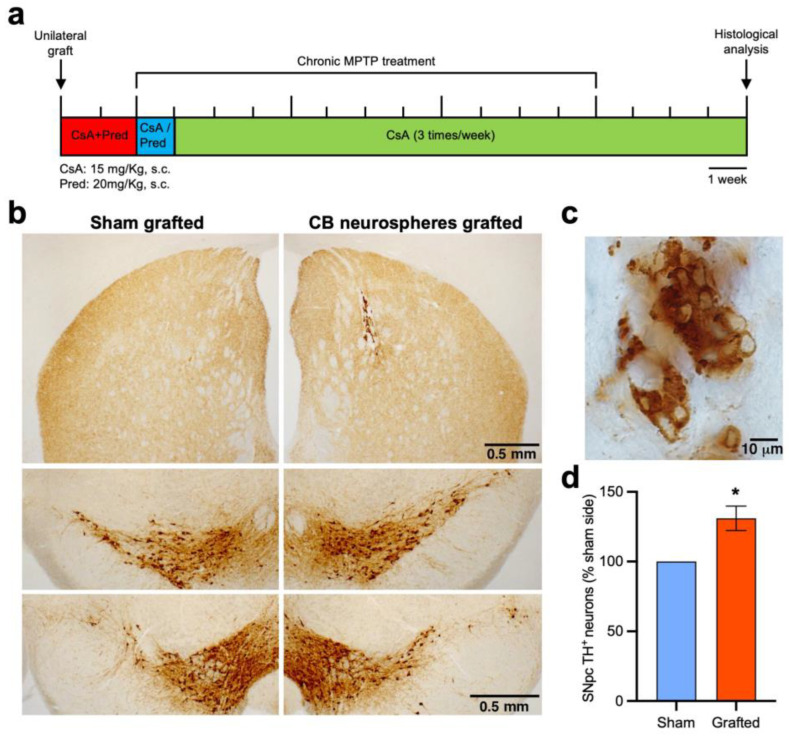
Protection of dopaminergic SNpc neurons through striatal xenografts of CB stem-cell-derived glomus cells. (**a**) Scheme of the experimental protocol used to analyze the neuroprotective effect of in vitro-expanded rat CB cells on mice dopaminergic nigrostriatal neurons. (**b**) Brain coronal sections, after TH immunohistochemistry, showing the sham (left panels) and CB-neurospheres-grafted (right panels) hemispheres at the level of striatum (top) and SNpc (middle and bottom). (**c**) Image, at higher magnification and after TH immunostaining, of the CB stem-cell-derived cells grafted in the striatum, showing an abundant number of in vitro-expanded dopaminergic glomus cells. (**d**) Stereological quantification, expressed as percentage of sham-grafted side, of ipsilateral TH^+^ SNpc neurons to the grafted striatum. Paired Wilcoxon test; *n* = 5; * *p* < 0.05.

The potential use of stem-cell-derived CB glomus cells in antiparkinsonian cell therapy was analyzed using in vitro rat CB-derived neurospheres, which provide higher yields of differentiated TH^+^ glomus cells than mouse CB neurospheres (see Figure 1a,b). Rat CB neurospheres were grafted in chronic MPTP parkinsonian mice [19,37] subjected to an immunosuppressive protocol developed by our group that allowed us to study the long-term effects of neural xenografts [31]. In a first assay, termed the “neuroprotection experiment”, we performed unilateral xenografts of rat CB neurospheres, with a sham graft in the contralateral hemisphere as internal control, on receptor mice that were subjected to immunosuppression (see the Materials and Methods Section and experimental scheme in Figure 2a). Two weeks later, xenografted mice were rendered parkinsonian through the chronic administration of MPTP (20 mg/Kg s.c.; three times/week for 3 months) and were allowed to recover from the toxic treatment for 1 month. Afterwards, the CB xenografted mice were euthanized and a histological examination of the SNpc TH^+^ neurons was carried out. In addition, to validate the degeneration of dopaminergic SNpc neurons produced by the chronic MPTP treatment, an experimental group of saline-treated mice was included. The histological analysis of the striatum revealed five animals with well-preserved intrastriatal grafts and with abundant in vitro-expanded differentiated TH^+^ dopaminergic CB glomus cells (Figure 2b,c). At the level of mesencephalon, the analysis of the SNpc of these animals revealed an important neuronal death of dopaminergic neurons in the sham-grafted side compared to the saline-treated mice (Table 1). However, the ipsilateral SNpc to the CB neurosphere graft showed a significantly higher number of TH^+^ nigral neurons with respect to the contralateral sham-grafted hemisphere (Table 1 and Figure 2b,d), indicating the trophic protection induced by the grafted CB glomus cells over these neurons.

**Figure 3:**
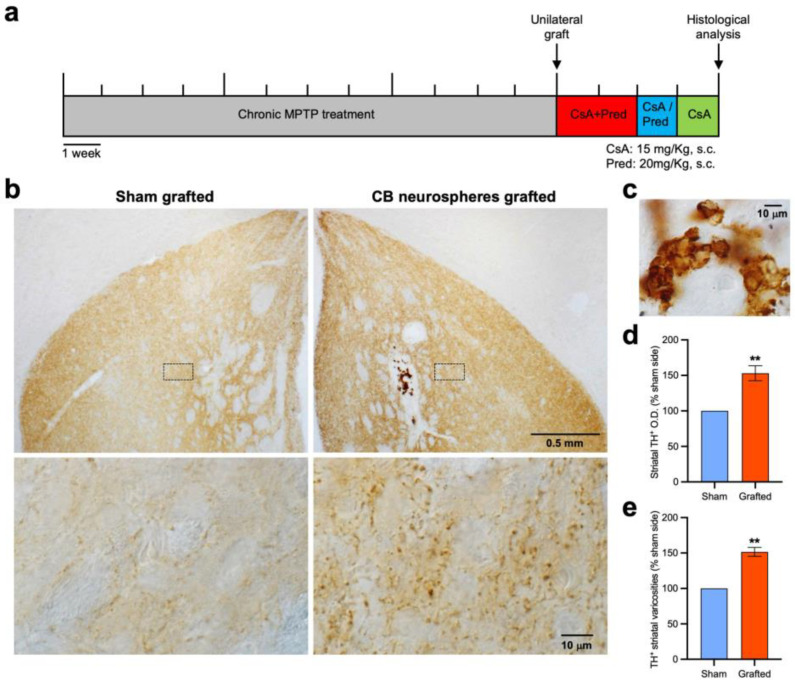
Restorative effects of CB neurospheres xenografts on dopaminergic striatal fibers. (**a**) Scheme of the experimental protocol used to test the reparative effect of in vitro-expanded rat CB cells on mouse dopaminergic striatal terminals. (**b**) Brain coronal sections, after TH immunohistochemistry, of the sham- (left) and CB-neurospheres-grafted (right) striata (upper panels). Insets depicted in lower panels illustrate the repair through axonal sprouting of the dopaminergic striatal terminals induced by the implant. (**c**) Image, at higher magnification and after TH immunostaining, showing the graft of CB stem-cell-derived TH^+^ glomus cells with abundant number of in vitro-expanded dopaminergic glomus cells. (**d**) Analysis of striatal dopaminergic innervation through measurements of TH^+^ optical density (O.D.). (**e**) Stereological quantification of dopaminergic striatal varicosities. In (**d**,**e**), data are expressed as percentage of sham-grafted side. Paired Wilcoxon test; *n* = 8; ** *p* < 0.01.

We also studied whether the transplantation of CB neurospheres could exert neuroregenerative actions (e.g., axonal sprouting) on a previously damaged dopaminergic striatal innervation. In this assay, termed the “restoration experiment”, receptor mice were subjected to the chronic MPTP treatment for three months, and once the toxic treatment was finished and the irreversible damage on nigrostriatal dopaminergic neurons induced [19,37], xenografts with rat CB neurospheres were performed. Xenografted CB mice were immunosuppressed after the transplantation and euthanized four weeks later for the histological examination (see experimental scheme in Figure 3a). All mice analyzed under this experimental design (*n* = 8) revealed well-maintained xenografts with numerous dopaminergic CB glomus cells (Figure 3b,c). As expected, in the sham-grafted striatum the chronic MPTP treatment induced an important decrease in dopaminergic innervation, revealed with measurements of the TH^+^ optical density (O.D.; saline, 19.69 ± 1.11 arbitrary units (a.u.) vs. the sham-grafted MPTP, 11.25 ± 0.71 a.u.; *p* < 0.0001) or through the stereological analysis of TH^+^ striatal varicosities (Table 1). Remarkably, the striatal xenografts of the in vitro-expanded CB glomus cells produced a significant increase in the dopaminergic striatal innervation in comparison with the contralateral sham-grafted hemisphere (Table 1 and Figure 3b,d,e). These results demonstrated that the striatal transplantation of CB neurospheres can induce an outgrowth (sprouting) of dopaminergic striatal fibers. Taken together, the results obtained both in the neuroprotection and restoration experiments clearly indicated that stem-cell-derived CB glomus cells maintained a similar neurotrophic potential as native CB tissue, inducing trophic protection and the restoration of the nigrostriatal pathway after the striatal transplantation.

### 2.3. Survival of Stem-Cell-Derived CB Glomus Cell Intrastriatal Transplants

A characteristic of CB glomus cells of relevance for neural transplantation is their extraordinary long survival in cerebral implants. This fact has been attributed to the physiological resistance of CB glomus cells to hypoxia [18,20,26,30], a condition present in intracerebral grafts [38]. In this work, we studied the survival of the xenografts of in vitro-generated TH^+^ CB glomus cells after an intrastriatal transplantation. In the first analysis, xenografts of CB neurospheres were performed in immunosuppressed mice that were not treated with MPTP, and were euthanized for the histological examination (*n* = 7) at different timepoints (between 2 and 12 weeks). In this analysis, all the grafts remained alive with numerous TH^+^ glomus cells, showing a similar survival as previously described for xenotransplants of native CB tissue [31]. In the case of the neuroprotection experiment (see Figure 2a), in which the CB neurospheres xenografts were performed before the chronic MPTP treatment, only 6 xenografts (1 located in the cortex and 5 located in the striatum) of the 32 grafted mice survived (~20% survival). In contrast, in the restoration experiment (see Figure 3a), where the transplants were carried out once the chronic MPTP treatment was finished and the dopaminergic toxin was washed out, 9 xenografts (1 located in the cortex and 8 in the striatum) of 11 grafted mice remained alive (~82% survival). The low survival of the CB neurospheres xenotransplants observed in the neuroprotection experiment seemed to be related to a toxic effect of the MPTP treatment on the grafted in vitro-expanded TH^+^ glomus cells, because high survival rates were observed in the other two experiments, in which grafted cells were not exposed to the neurotoxin. Moreover, the six xenografts that survived in the neuroprotection experiment had abundant and apparently healthy TH^+^ CB cells, even five months after transplantation, suggesting that not all in vitro cultures of CB neurospheres had the same degree of susceptibility to the dopaminergic toxin.

## 3. Discussion

CB cell therapy has been proven to be effective in rodent and nonhuman primate preclinical PD models [16,17,18,19,31,39,40,41]. The analyses of the mechanism underlying the antiparkinsonian action of CB transplants revealed that their beneficial effect was due to a trophic stimulation of the nigrostriatal pathway, mainly mediated by GDNF released by grafted CB glomus cells [18,19,21]. Furthermore, two pilot phase I/II open trials evaluated the feasibility, safety and clinical efficacy of CB autotransplantation in PD patients [23,24], showing that it is a safe and feasible procedure that produces a clinical improvement in moderately affected patients, similar to that obtained after hfVM grafts [6,7]. However, the effectiveness of CB transplantation observed in PD patients was shown to be considerably lower than in experimental models. Among the factors that could influence the clinical outcome of CB cell therapy, the scarcity of the grafted tissue appears as a principal one [24]. In this work, we evaluated the potential use of in vitro-generated dopaminergic CB glomus cells through the culture and differentiation of neural stem cells present in the organ, as a way to increase the amount of tissue available for grafting in PD patients. Previous reports demonstrated that stem-cell-derived CB glomus cells have a highly differentiated dopaminergic phenotype, expressing voltage-gated Ca^2+^ and K^+^ channels and secreting catecholamines in response to hypoxia or hypoglycemia as mature glomus cells [27,28,42]. Here, we also showed that in vitro-generated TH^+^ glomus cells have similar neurotrophic features to native CB glomus cells, producing comparable amounts of GDNF and maintaining the expression of the neurotrophic factor after a striatal transplantation. The generation of differentiated dopaminergic CB glomus cells through the in vitro culture of CB neural stem cells has been described in rats, mice and humans [27,28,29,43]. However, our study revealed important variations in terms of proliferation and differentiation in TH^+^ CB glomus cells between rats and mice, with rat neurospheres being much larger and having more dopaminergic cells than mouse neurospheres. Additional experimental work should be performed to identify the mechanisms of the proliferation and differentiation of CB stem cells, in order to increase the yield of in vitro-expanded CB dopaminergic glomus cells for their use in antiparkinsonian cell therapy.

The analysis of the survival of stem-cell-derived CB glomus cell intracerebral implants revealed two relevant findings. On the one hand, striatal xenografts of in vitro-generated TH^+^ CB glomus cells showed similar rates of survival (~80–100%) as previously reported for both xeno- and allotransplantations of native CB cells [18,19,31]. On the other hand, cerebral implants of in vitro-generated CB glomus cells presented an elevated susceptibility to the dopaminergic neurotoxin MPTP, an observation that contrasts with the resistance reported for native CB tissue [19,21,31]. The factors that determine this high susceptibility of stem-cell-derived CB glomus cells to MPTP are unknown, and should be addressed in future experimental studies. It is also relevant to note that we did not see signs of tumorigenicity or other deleterious effects in any of the grafted mice at the different timepoints studied (2, 4, 5, 12 and 18 weeks), indicating the safety of the intracerebral grafting of in vitro-generated dopaminergic CB glomus cells.

To study whether the transplants of stem-cell-derived CB glomus cells could induce neurotrophic protection and restoration (axonal sprouting) of damaged dopaminergic nigral neurons, we used a methodology previously validated for striatal grafts of native CB cells [19,31]. Remarkably, the neuroprotective and restorative effects exerted by the xenografts of in vitro-generated dopaminergic CB cells were similar to those previously described for allografts [19] or xenografts [31] of native CB cells in the chronic MPTP parkinsonian model. The fact that the immunosuppressive protocol used did not alter the antiparkinsonian effects of stem-cell-derived CB glomus cells opens the possibility to obtain in vitro-generated human CB glomus cells not only from PD patients, through the unilateral resection of the organ, but also from allogenic donors. In conclusion, the data presented here clearly showed that stem-cell-derived CB glomus cells have similar neurotrophic features to native tissue. Stem-cell-derived dopaminergic and GDNF-producing glomus cells represent a new therapeutic option to be considered in cell therapy in PD. These cells could be useful either for inducing neurotrophic protection and the stimulation of damaged SNpc neurons or for improving the survival and efficacy of hfVM transplants [9,44,45]. The neurotrophic potential of stem-cell-derived CB glomus cells could also be relevant for the treatment of other neurodegenerative diseases [46,47].

## 4. Materials and Methods

### 4.1. Animal Care and Pharmacological Treatments

C57BL/6N and heterozygous GDNF/lacZ mice of 2-3 months of age (CEA-Oscar Pintado, University of Seville) were housed in a temperature-regulated environment (22 ± 1 °C) on a 12 h light/dark cycle with ad libitum access to food and water. The mice were rendered parkinsonian as previously described [19,31,37] through the subcutaneous (s.c.) administration of MPTP (20 mg/kg; Sigma, St. Louis, MO, USA) 3 times per week for 3 months. An immunosuppressive treatment was applied to the xenografted mice as previously indicated [31]. Briefly, animals received daily injections of cyclosporine A (CsA; 15 mg/Kg; Sigma; s.c.) and prednisone (Pred; 20 mg/Kg; Sigma; s.c.) during the first two weeks, alternating daily injections of CsA (15 mg/Kg; s.c.) or Pred (20 mg/Kg; s.c.) for 1 week and 3 doses per week of CsA (15 mg/Kg; s.c.) until they were euthanized for the histological analysis. At the end of the experiments, the mice were euthanized under deep anesthesia induced with a combination of 100 mg/kg ketamine (Pfizer, New York, NY, USA) and 10 mg/kg xylazine (Bayer, Leverkusen, Germany). All experiments were carried out according to the European Directive 2010/63/EU and the Spanish RD/53/2013 for the protection of animals used for scientific purposes. The animal procedures were approved by the Animal Research Committee of the University Hospital Virgen del Rocío (University of Seville; registered agreement number 11-07-14-112).

### 4.2. CB Neurosphere Cultures

Rat and mouse CB cell dissociation and neurospheres assays were carried out as described by our laboratory [27,28,35,48]. CBs were dissociated using an enzymatic treatment with 0.6 mg/mL collagenase type II (Sigma, St. Louis, MO, USA), 0.3 mg/mL trypsin (Sigma) and 5 mg/mL porcine pancreas elastase (Calbiochem, San Diego, CA, USA) in a PBS solution and 50 μM CaCl_2_ for 20 min at 37 °C in a crystal flask at 600 rpm in a Thermomixer Comfort (Eppendorf, Hamburg, Germany). After that, 2 volumes of blocking solution (for 50 mL: 44 mL L15 medium (GIBCO, Waltham, MA, USA), 0.5 mL penicillin/streptomycin (GIBCO), 0.5 mL 1 M HEPES buffer (GIBCO), 2.5 mL FBS 5% (GIBCO) and 2.5 mL distilled and deionized water) were added to quench the enzymes. Dissociated CB cells were centrifuged for 5 min at 300× *g* at 4 °C and the cell pellet was resuspended and plated on ultralow binding 6-well plates (Corning, New York, NY, USA) at a clonal density of 10.000 cells per well. The culture medium contained D-MEM:F-12 (GIBCO) with 15% FBS (GIBCO), 1% N2 supplement (GIBCO), 2% B27 supplement (GIBCO), 1% penicillin/streptomycin (GIBCO), 20 ng/mL recombinant human bFGF (R&D Systems, Minneapolis, MN, USA), 20 ng/mL recombinant human IGF-1 (R&D Systems) and 20 ng/mL recombinant human EGF (R&D Systems). All cultures were maintained in O_2_- and CO_2_-controlled incubators (Thermo, Waltham, MA, USA) at 3% and 5% respectively and 37 °C for 10–12 days before being used for grafting.

### 4.3. CB Neurosphere Xenografting

Mice grafted with rat CB neurospheres xenografts received a CsA injection (15 mg/kg; s.c.) 4–6 h before starting the surgical procedure. An intrastriatal graft of CB neurospheres was performed as previously described for native CB tissue [19,31]. Briefly, 100–150 CB neurospheres were placed in 1 μL of Tyrode’s solution (140 mM NaCl; 4.7 mM KCl; 2 mM CaCl_2_; 1 mM MgCl_2_; 10 mM 4-2-hydroxyethyl-1-piperazineethanesulfonic acid, HEPES; Sigma; in distilled and deionized water) and were stereotaxically injected into the striatum (from bregma in mm: anteroposterior, +0.4; lateral, +2; ventral, −3.5) with a 25-gauge syringe (Hamilton, Bonaduz, Germany) according to the mouse brain stereotaxic atlas [49]. As an internal control, a sham graft was performed in the contralateral striatum, injecting 1 μL of the vehicle solution. To avoid differences between animals, stereologic and densitometric values (see below) of CB-neurosphere-grafted parkinsonian mice were expressed as a percentage of the sham-grafted hemisphere.

### 4.4. Histological Analyses

Mice were transcardially perfused with 50 mL of PBS (Sigma) and 50 mL of 4% paraformaldehyde (Sigma) in PBS. The brains were immediately removed and fixed overnight at 4 °C with 4% paraformaldehyde in PBS. After fixation, the brains were cryoprotected in 30% sucrose (Sigma) in PBS and included in an optimum cutting temperature compound (O.C.T. compound, Tissue-Tek, Tokyo, Japan). The neurospheres were fixed in 4% paraformaldehyde in PBS for 20 min, washed 3 times in PBS for 5 min each, cryoprotected in 30% sucrose for 30 min and, finally, embedded in the OCT compound and frozen. Brain coronal sections (30 μm thickness) or embedded neurospheres (10 μm thickness) were cut on a cryostat (Leica, Wetzlar, Germany). Tyrosine hydroxylase (TH) and nestin immunodetection was performed as previously described [19,31,48] using, respectively, polyclonal anti-TH (1:1000; Novus Biologicals, NB300-109), monoclonal anti-nestin (1:500; Merck-Millipore, Burlington, MA, USA, MAB353) and a secondary peroxidase-conjugated antibody kit (Dako, Hovedstaden, Denmark) or fluorescence secondary antibodies (goat–antirabbit Alexa568, 1:400, Invitrogen; goat–antimouse Alexa488, 1:400, Jackson ImmunoResearch, West Grove, PA, USA). The image acquisition and analysis were performed with light transmitted (AX70 or Bx61, both with digital refrigerated camera DP72, Olympus, Tokyo, Japan) and their specific imaging software (CellSens v 1.4.1).

### 4.5. GDNF ELISA

The GDNF protein content was measured using the methodology described before by our group [21,44]. Briefly, rat SCG, CB and CB neurospheres were frozen in liquid N_2_ and homogenized in a lysis buffer (137 mM NaCl; 20 mM Tris, pH 8.0; 1% IGEPAL CA-630, 10% glycerol, 1/1000 protease inhibitor cocktail; Sigma) using a polytron (OMNI, Waterbury, CT, USA). Protein extraction and the ELISA assay were performed following the manufacturers’ instructions (G7621, Promega, Madison, WI, USA), except for the anti-GDNF monoclonal and anti-hGDNF polyclonal antibodies, which were used at 1:500 and 1:250, respectively. The absorbance at 450 nm was measured in a plate reader (Thermo) and the total protein content of the samples was obtained using the Bradford assay (Bio-Rad, Hercules, SA, USA).

### 4.6. Stereology and Densitometry

Unbiased stereological analyses were performed with the use of systematic random sampling using the optical dissector method [50]. All stereological procedures were performed using the C.A.S.T. grid system (Olympus) or new CAST^TM^ system (Visiopharm, Hørsholm, Denmark), following the methodology described by our laboratory [19,31,37]. Estimations of TH^+^ SNpc neurons and striatal dopaminergic varicosities were carried out in the regions spanning from −3.08 mm to −3.28 mm (SNpc) and from 0.80 mm to 0.10 mm (striatum), relative to Bregma, according to the Franklin and Paxinos mouse brain stereotaxic atlas [49]. Only SNpc cells lateral to the medial terminal nucleus of the accessory optic tract were determined to have a clear separation from the adjacent ventral tegmental area [51]. Reference volumes for each section were outlined under low magnification (4×), and TH^+^ neurons or varicosities were counted at high magnification (40×) using, respectively, 6417.3 μm^2^ × 20 μm or 114.1 μm^2^ × 20 μm optical dissectors, with a guard volume of 5 μm to avoid artefacts on the cut surface of the sections. The optical density (O.D.) of the striatal TH^+^ innervation was measured from digitized pictures using the NIH Image software (ImageJ, v 13.0.6, Bethesda, MD, USA), as previously described [31,37]. The optical density values of each animal were obtained from the entire rostrocaudal extent of the same striatal region analyzed through unbiased stereology.

### 4.7. Statistical Analysis

The number of mice analyzed in each experimental group and the statistical tests applied were indicated in each figure/table legend. Data were presented as the mean ± standard error of the mean (SEM). In all cases, normality and equal variance tests were performed and, when passed, a *t*-test (unpaired and paired) was carried out. In the cases where normality or homoscedasticity tests failed, the nonparametric Kruskal–Wallis H with post hoc Dunn’s test, Mann–Whitney or paired Wilcoxon tests were performed. All statistical analyses were conducted using Prism 9.0 (GraphPad Software, Boston, MA, USA).

## Figures and Tables

**Table 1:** Stereological analysis of dopaminergic nigral neurons and striatal varicosities.

Neuroprotection Experiment	*n*	TH^+^ SNpc Neurons
Saline Sham-grafted MPTP CB-neurospheres-grafted MPTP	4 5 5	3148 ± 81.45 * (*p* = 0.015) 1935 ± 277.90 2496 ± 330.50 * (*p* = 0.031)
Restoration Experiment	*n*	TH^+^ Str Varicosities (10^6^/mm^3^)
Saline Sham-grafted MPTP CB-neurospheres-grafted MPTP	6 8 8	12.95 ± 0.37 **** (*p* < 0.0001) 7.37 ± 0.25. 11.14 ± 0.48 **** (*p* < 0.0001)

* *p* < 0.05 (saline vs. sham-grafted MPTP, Mann–Whitney test; sham-grafted MPTP vs. CB-neurospheres-grafted MPTP, paired Wilcoxon test). **** *p* < 0.0001 (saline vs. sham-grafted MPTP, unpaired *t*-test; sham-grafted MPTP vs. CB-neurospheres-grafted MPTP, paired *t*-test).

## Data Availability

All relevant data were included in the paper. This study did not generate datasets deposited in external repositories. Information/data required can be made available by the corresponding author upon request.
